# Evolution and suicide: critique of the pain and brain model

**DOI:** 10.1192/bjb.2024.93

**Published:** 2025-04

**Authors:** Riadh Abed, Paul St John-Smith

**Affiliations:** 1 Mental Health Tribunals, Ministry of Justice, UK; 2 Hertfordshire Partnership University NHS Foundation Trust, UK

**Keywords:** Suicide, evolutionary psychiatry, pain and brain, Darwinian theory, suicidality

## Abstract

Soper's ‘pain and brain’ evolutionary theory of suicide has significant explanatory power and deserves wider consideration and scrutiny in the mainstream psychiatric literature. It provides a novel framework for thinking about the problem of suicide and could have an important impact on research as well as clinical practice. However, we raise questions and concerns regarding the prediction the theory makes regarding common mental disorders being anti-suicide adaptations.

The short article by Swanepoel and Soper in this issue, titled ‘Mental disorders may prevent, not cause, suicide’,^1^ is an eloquent exposition of Soper's ‘pain and brain’ evolutionary model of suicide. Although the pain and brain model is well-known within evolutionary circles and is, in our view, the most persuasive evolutionary theory of suicide in the current literature, it has not received adequate attention or scrutiny by mainstream psychiatrists. We therefore consider it timely and commendable that *BJPsych Bulletin* has taken the lead in introducing Soper's model to a wider psychiatric readership. The pain and brain model illustrates the power of evolutionary theory in organising an array of disparate facts through providing an explanatory framework that can help in the understanding of a complex human phenomenon such as suicide. An evolutionary framework can help us think more clearly about the nature of a given biological phenomenon, and provide the basis for future fruitful theories and hypotheses. However, besides the ability to explain what we already know, it is important that evolutionary models (and scientific theories generally) make testable and falsifiable predictions about what is currently unknown, and this is what the authors have attempted.

The pain and brain model of suicide builds on the idea that during the evolution of our species, the human cognitive capacity, unlike all other organisms on earth, crossed a processing threshold that uniquely enabled the human organism to evaluate their possible level of future suffering, comprehend the possibility and effectiveness of ending one's own suffering by self-killing, and to know technically how to achieve that end. The model predicts that the crossing of this threshold had such potentially devastating effects on fitness (the chances of survival and reproduction) that survival (in the suicide niche^2^) would not have been possible had natural selection not shaped adaptations that prevented such an outcome: anti-suicide adaptations. These are predicted by the model to exist in multiple layers. Furthermore, the model predicts that natural selection has had enough time to reduce the risk of suicide down to a level where it is exceedingly difficult, if not impossible, for human intervention to improve on. This is why the authors suggest that suicide prediction in any given individual is unreliable and possibly no better than chance. The evidence appears to support this. Thus, the pain and brain model is uniquely able to account for a range of facts and observations around suicide, which include why it is a species-specific human phenomenon, why it is exceedingly rare before 10 years of age, why it is absent in those with severe intellectual disabilities and that natural selection would have shaped a range of suicide prevention adaptations. Up to this point in the model, many evolutionists would agree with the pain and brain model.

However, what the authors go on to propose is that the final line of defence against suicide, designed by natural selection, includes most forms of common mental disorders (CMDs), with some notable exclusions. The exclusions include childhood mental disorders, organic brain disease and neurodevelopmental disorders. Anxiety disorders were also excluded on the basis that they form a distinct category of conditions that stem from phylogenetically ancient defence mechanisms that are shared across many species.^3^ The extension of the pain and brain model to include the claim that the majority of CMDs are anti-suicide adaptations is a bold claim that is both controversial and potentially problematic.

We recognise that although counter-intuitive claims may be hard to accept, they are not necessarily wrong. However, such a claim must be supported by empirical evidence, one might argue, particularly strong evidence, commensurate with its wide scope and profound implications. How can this be resolved? First, contradictory evidence must also be sought, not just confirmatory evidence for this theory. Situations and evidence must be investigated that could refute the idea as a generalised process. Second, several other logical options need consideration. Do CMDs prevent suicide? Or as conventional understanding has it, does mental disorder cause suicidality? Are they just two phenomena that often occur together, are there other confounding issues and is there a predictive mathematical relationship – if so, what? And in which direction? Are there other options such as another sequence of events; for instance, could a social or environmental cause like a loss, explain both phenomena together?

The Bradford Hill criteria^4^ (BHC) could help us as a guide. These are a set of principles that can help in evaluating epidemiological evidence of a causal relationship between a presumed cause and an observed effect, and have been widely used in public health research. The criteria include strength (effect size), consistency (reproducibility), specificity, temporality, dose–response relationship, plausibility and coherence.

An additional criterion, that of reversibility, the converse of temporality, may be relevant in this particular case; so that if the cause is removed (suicidality), would the effect (CMD) disappear?

Thus, although the contention that all CMDs have a common anti-suicide origin is consistent with unitary models of mental disorder that propose the existence of a common factor underlying all CMDs (sometimes referred to as the ‘P’ factor^5^) as well as with the high levels of comorbidity and overlapping aetiology, we suggest there is a range of counter-evidence that calls for a somewhat cautious and circumspect approach. We briefly discuss some theoretical difficulties and counter-evidence to this contention.

## Mental disorder as protection against suicide

One possible issue to consider is that mental distress (psychic pain) in a sufferer elicited a generic care by others. So, the argument that may be put forward is that *some* CMDs may have evolved to elicit care from others, and, hence, care may have been at the root of their anti-suicide effect. Thus, evaluating the proposal that CMDs are suicide prevention adaptations within the BHC framework raises the problems of coherence and plausibility, as well as direct causality.

## The male preponderance in both suicide and severe mental illness

The estimated male:female ratio of suicide across the globe was 2.33:1,^6^ with much of the world outside China showing a much higher male preponderance of suicide. The highest level of female suicide internationally was just above 10 per 100 000 population, whereas the highest male suicide rate exceeded 45 per 100 000 population.^6^ According to Swanepoel and Soper, lower suicide rates must be accompanied by higher CMDs, but the evidence for this is either unclear or pointing toward males suffering higher rates of severe CMDs, especially when psychotic symptoms are considered. The mental disorders that show clear female preponderance include anxiety and depression (mild to moderate depression) and eating disorders. However, as stated previously, Soper^3^ has excluded anxiety from being an anti-suicide adaptation, and it is noted that although moderate depression shows a female preponderance, the more severe psychotic depression affects genders more equally.^7^ This is a further problem for the prediction, as the more severe forms of the CMD should have greater suicide prevention potential because of greater impairment of concentration, motivation and energy. Furthermore, all severe psychotic disorders are either gender equal or have a male preponderance (e.g. schizophrenia has a male:female ratio of around 1.4:1^8^), and this is reflected in the statistics for in-patient admissions internationally.^9^ For example, although out-patient visits were very similar for men and women (52% male versus 48% women), the percentage of male in-patient admissions across the world ranged from 56 to 69%.^9^ If Swanepoel and Soper's prediction is correct, the data on male preponderance both on suicide and severe mental disorder raises the question as to how natural selection has managed to reduce the suicide rates in females without the enormously high cost of increased severe and debilitating CMDs?

Although Swanepoel and Soper's proposal that the lower female suicide rates can be explained by the use of less lethal methods (overdosing and cutting), where females show a large preponderance of self-harm behaviour,^10^ this is inconsistent with the epidemiological data that shows a consistently low level of completed suicides in females despite a large peak of self-harm behaviour in the late teens and early 20s.^10^ Within the BHC, these raise the problems of plausibility, biological gradient and consistency of the model. However, the idea that self-harm itself may be an anti-suicide strategy has some empirical support.^11^

## The lack of evidence of increased risk of severe mental illness in severe chronic physical pain

As the authors have stated, testing the prediction that severe chronic physical pain is a risk factor for CMDs, including severe (psychotic) illness, may be a feasible way of testing the model. However, from what we know already regarding chronic physical pain, this is likely to be a serious challenge to the authors’ contention. Other than anxiety and depression (without psychotic symptoms), there are no current reports or observations of increased risk of severe psychotic mental illness in this population. Given the ubiquity of pain clinics around the world, there would have been some indication in the current literature of such an increased risk or association, but, to our knowledge, this is not the case. Within the BHC, this is a problem for the consistency, specificity, coherence and plausibility of the prediction that pain leads to suicidality which leads to the activation of suicide prevention adaptations in the form of (severe) CMDs.

## Human mood and optimism as suicide prevention adaptations

According to the pain and brain model, the first line of defence against suicide is the human trait of ‘warmer than neutral mood’.^12^ This is the psychobiological basis for the human capacity for maintaining unrelenting optimism in the face of adversity (and psychic pain) and the ability to battle on against seemingly impossible odds. Yet, according to the model, this evolved human suicide prevention state is ‘switched off’ in depression, to be replaced by a state of ‘emotional numbness’, where both psychic pain as well as pleasure are dulled. Although it is plausible that a state of moderate to severe, severe, melancholic and psychotic depression can seriously interfere with an individual's capacity to plan and execute the act of suicide (through emotional numbness, drained energy and impaired cognitive functioning), it is not at all clear how this can work in mild to moderate depression. In mild to moderate depression, there is both a switching off of the evolved ‘warmer than neutral’, optimistic anti-suicide state while energy and cognitive capacities are relatively preserved. And it is worth noting that it is these less severe and less disabling depressive states that are more common in women, who have a lower risk of completed suicide. Thus, according to the BHC, questions are raised regarding the plausibility of emotional numbness as being a more effective anti-suicide adaptation than the ‘warmer than neutral mood’ state in mild to moderate depression.

In addition to the above three areas of concern, there are a number of other challenges that can be levelled against the proposal that CMDs are suicide prevention measures. These include the fact that there is tremendous variation in levels of chronicity of CMDs, which range from brief single episodes to lifelong chronic afflictions, and this would be difficult to accommodate in a single causal model.

## Conclusions

We suggest that the pain and brain evolutionary model of suicide shows considerable promise and deserves greater attention from mainstream psychiatry and psychology. However, like the rest of evolutionary psychiatry, its explanatory power relies on empirical evidence. Although several elements of the model meet multiple criteria of causation, the prediction that the majority of CMDs are evolved defences against suicide is, in our view, the least supported part of the model. It is possible that we may be proved wrong through well-designed empirical studies, but as things stand, the range of counterevidence does not, in our opinion, currently support this sweeping prediction. However, we hope that a re-examination of suicide in the light of the pain and brain model can generate important and novel insights into this serious and tragic human phenomenon.

## About the authors

**Riadh Abed,** FRCPsych, is a consultant psychiatrist with the Mental Health Tribunals of the Ministry of Justice, London, UK. **Paul St John-Smith,** FRCPsych, is a retired consultant psychiatrist at Hertfordshire Partnership University NHS Foundation Trust, St Albans, UK.
